# Structure Modeling of All Identified G Protein–Coupled Receptors in the Human Genome

**DOI:** 10.1371/journal.pcbi.0020013

**Published:** 2006-02-17

**Authors:** Yang Zhang, Mark E DeVries, Jeffrey Skolnick

**Affiliations:** Center of Excellence in Bioinformatics, University at Buffalo, Buffalo, New York, United States of America; Cornell University, United States of America

## Abstract

G protein–coupled receptors (GPCRs), encoded by about 5% of human genes, comprise the largest family of integral membrane proteins and act as cell surface receptors responsible for the transduction of endogenous signal into a cellular response. Although tertiary structural information is crucial for function annotation and drug design, there are few experimentally determined GPCR structures. To address this issue, we employ the recently developed threading assembly refinement (TASSER) method to generate structure predictions for all 907 putative GPCRs in the human genome. Unlike traditional homology modeling approaches, TASSER modeling does not require solved homologous template structures; moreover, it often refines the structures closer to native. These features are essential for the comprehensive modeling of all human GPCRs when close homologous templates are absent. Based on a benchmarked confidence score, approximately 820 predicted models should have the correct folds. The majority of GPCR models share the characteristic seven-transmembrane helix topology, but 45 ORFs are predicted to have different structures. This is due to GPCR fragments that are predominantly from extracellular or intracellular domains as well as database annotation errors. Our preliminary validation includes the automated modeling of bovine rhodopsin, the only solved GPCR in the Protein Data Bank. With homologous templates excluded, the final model built by TASSER has a global C_α_ root-mean-squared deviation from native of 4.6 Å, with a root-mean-squared deviation in the transmembrane helix region of 2.1 Å. Models of several representative GPCRs are compared with mutagenesis and affinity labeling data, and consistent agreement is demonstrated. Structure clustering of the predicted models shows that GPCRs with similar structures tend to belong to a similar functional class even when their sequences are diverse. These results demonstrate the usefulness and robustness of the in silico models for GPCR functional analysis. All predicted GPCR models are freely available for noncommercial users on our Web site (http://www.bioinformatics.buffalo.edu/GPCR).

## Synopsis

G protein–coupled receptors (GPCRs) are a large superfamily of integral membrane proteins that transduce signals across the cell membrane. Because of the breadth and importance of the physiological roles undertaken by the GPCR family, many of its members are important pharmacological targets. Although the knowledge of a protein's native structure can provide important insight into understanding its function and for the design of new drugs, the experimental determination of the three-dimensional structure of GPCR membrane proteins has proved to be very difficult. This is demonstrated by the fact that there is only one solved GPCR structure (from bovine rhodopsin) deposited in the Protein Data Bank library. In contrast, there are no human GPCR structures in the Protein Data Bank. To address the need for the tertiary structures of human GPCRs, using just sequence information, the authors use a newly developed threading-assembly-refinement method to generate models for all 907 registered GPCRs in the human genome. About 820 GPCRs are anticipated to have correct topology and transmembrane helix arrangement. A subset of the resulting models is validated by comparison with mutagenesis experimental data, and consistent agreement is demonstrated.

## Introduction

G protein–coupled receptors (GPCRs) are integral membrane proteins embedded in the cell surface that transmit signals to cells in response to stimuli such as light, Ca^2+^, odorants, amino acids, nucleotides, peptides, or proteins and mediate many physiological functions through their interaction with heterotrimeric G proteins [1,2]. Many diseases involve the malfunction of these receptors, making them important drug targets. In human, the estimated number of GPCRs is approximately 948 [3], corresponding to about 5% of the total number of human genes [4]. However, more than 45% of all modern drugs target GPCRs; these represent around 25% of the 100 top-selling drugs worldwide [2,5].

While knowledge of a protein's structure furnishes important information for understanding its function and for drug design [6], progress in solving GPCR structures has been slow [7]. Nuclear magnetic resonance (NMR) spectroscopy and X-ray crystallography are the two major techniques used to determine protein structures. NMR spectroscopy has the advantages that the protein does not need to be crystallized and dynamical information can be extracted. However, high concentrations of dissolved proteins are needed; and as yet no complete GPCR structure has been solved by the method. X-ray crystallography can provide very precise atomic information for globular proteins, but GPCRs are extremely difficult to crystallize. In fact, only a single GPCR, bovine rhodopsin (RH) from the rod outer segment membrane, has been solved [8]. It is unlikely that a significant number of high-resolution GPCR structures will be experimentally solved in the very near future. This situation limits the use of structure-based approaches for drug design and restricts research into the mechanisms that control ligand binding to GPCRs, activation and regulation of GPCRs, and signal transduction mediated by GPCRs [9].

Fortunately, as demonstrated by the recent CASP experiments [10], computer-based methods for deducing the three-dimensional structure of a protein from its amino acid sequence have been increasingly successful. Among the three types of structure prediction algorithms—homology modeling (comparative modeling [CM]) [11,12], threading [13,14], and ab initio folding [15–17]—CM, which builds models by aligning the target sequence to an evolutionarily related template structure, provides the most accurate models. However, its success is largely dictated by the evolutionary relationship between target and template proteins. For example, for proteins with greater than 50% sequence identity to their templates, CM models tend to be quite close to the native structure, with a 1-Å root-mean-squared-deviation (RMSD) from native for their backbone atoms, comparable to low-resolution X-ray and NMR experiments [12,18]. When the sequence identity drops below 30%, termed the “twilight zone,” CM model accuracy sharply decreases because of the lack of a significant structure match and substantial alignment errors. Here, the models provided by CM are often closer to the template on which the model is based rather than the native structure of the sequence of interest. This has been a significant unsolved problem [19]. Among all registered human GPCRs, there are only four sequences that have a sequence identity to bovine RH greater than 30%. Ninety-nine percent of human GPCRs, with an average sequence identity to bovine RH of 19.5%, lie outside the traditional comparative modeling regimen [9].

Recently [14,17,20,21], we developed the threading assembly refinement (TASSER) methodology, which combines threading and ab initio algorithms to span the homologous to nonhomologous regimens. In a large-scale, comprehensive benchmark test of 2,234 representative proteins from the Protein Data Bank (PDB) [22], after excluding templates having greater than 30% sequence identity to the target, two thirds of single domain proteins can be folded to models with a C_α_ RMSD to native of less than 6.5 Å [20,21]. As a significant advance over traditional homology modeling, many models (including membrane proteins) are improved with respect to their threading templates (858 of 2,234 targets have an RMSD improvement of greater than 1.5 Å).

In the absence of additional GPCR crystal structures, computer-based modeling may provide the best alternative to obtaining structural information [23–28]. In this work, we exploit TASSER to predict tertiary structures for all 907 GPCR sequences in the human genome that are less than 500 amino acids in length. Only the sequence of the given GPCR is passed to TASSER and no other extrinsic knowledge (e.g., active sites and binding regions, experimental restraints, etc.) is incorporated into our structure prediction approach. Because the rearrangements of TM helices from RH may occur for nonhomologous GPCRs, the ability to refine templates is the most important advantage of using TASSER in comprehensive GPCR modeling. Also, distinct from many other GPCR modeling methods that only attempt to model the TM helical regions [27,29,30], TASSER generates reasonable predictions for the loop regions. In benchmark tests [21], for 39% of loops of four or more residues, TASSER models have a global RMSD less than 3 Å from native. In contrast, using the widely used homology modeling tool, MODELLER [11,12], the percentage of loops with this accuracy is 12% [20]. If one considers only the accuracy of the loop conformation itself (and neglects its orientation relative to the remainder of the protein), then 89% of the TASSER*-*generated loops have a local RMSD of less than 3 Å, and the average RMSD for loops up to 50 residues is below 4 Å. This is especially important in GPCR modeling as the extracellular loops are often critical in determining ligand specificity [31–33]. Therefore, full-length TASSER models offer substantial advantages over traditional comparative modeling methods and are likely to be of greater aid in understanding the ligand and signaling interactions of GPCRs.

## Results

### Application of TASSER to Membrane Proteins

Two forms of TASSER were developed for this study that slightly differ from our previously published work [14,17,20,21]. The first form of TASSER was extended to explicitly model TM proteins by including a “hydrophilic inside” potential for predicted TM regions as described in Materials and Methods. Modeling bovine RH with this form of TASSER demonstrates the effectiveness of this approach. On excluding homologous structures whose sequence identity greater than 30%, PROSPECTOR_3 identified three templates, 1pv6B (lactose permease), 1b3uA (protein phosphatase 2A), and 1a8hA (methionyl-tRNA synthetase), with Z-scores of 8.1, 8.7, and 5.3, respectively; bovine RH is therefore assigned as a medium/hard target. After the TASSER simulation, 76% of the structures from the 14 lowest temperature replicas are found inside a cluster with an RMSD cutoff of 8 Å. The average RMSD of these structures to the cluster centroid is 4.2 Å, which gives a C-score of 0.45. Of targets with this score, 82% are foldable according to the PDB benchmark [20]. In Figure 1, we show the comparison of both threading templates and the model of highest structure density with respect to the crystal structure. An RMSD of 4.6 Å from native for the final model is obtained if we superimpose all 338 C_α_ atoms (ten residues are absent in the crystal structure). The major modeling errors are in the N- and C-termini and the C3 loop. If we excise the tails and superimpose the model onto the core region (residues 32 to 323) of the native structure, the RMSD between the model and native structure is 3.3 Å. When we consider only the TM helix region, that is, TM1 (35 to 64), TM2 (71 to 100), TM3 (107 to 139), TM4 (151 to 173), TM5 (200 to 225), TM6 (247 to 277), and TM7 (286 to 306), the RMSD is 2.1 Å.

**Figure 1:**
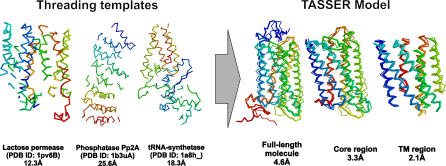
Initial Templates from PROSPECTOR_3 and the Final TASSER Model of Highest Cluster Density Superposed on the Bovine RH Crystal Structure Blue to red runs from N- to C-terminus. The numbers are the RMSD to native. Images are from RASMOL [120].

A second integrated form of TASSER was constructed that incorporates a TM potential but selectively applies it without prior knowledge as to whether a target sequence is a membrane protein. Application of this integrated potential to a benchmark set of 38 membrane proteins (excluding all templates with greater than 30% identity in the aligned region) results in 17 targets with an RMSD to native less than 6.5 Å and an average improvement over the template alignment of 4.9 Å with 97% of targets showing an improvement compared to the starting template (Figure 2). A detailed list of the threading templates and final model information for the 38 membrane proteins is presented at Table 1. It should also be noted that more than 60% of the structures in the benchmark were proteins crystallized as part of large heteroprotein complexes. Applying this to the four other known seven-TM proteins in the PDB database, archeorhodopsin (1uaz), sensory rhodopsin (1jgj), halorhodopsin (1e12), and bacteriorhodopsin (1ap9), yields final models with RMSDs to native of 2.66, 1.25, 2.39, and 1.86 Å, respectively (Table 1). In all cases, TASSER refined the starting template closer to native, with archeorhodopsin showing a change in RMSD from 19.78 Å for the highest scoring template to 1.22 Å over the same aligned region in the final model, sensory rhodopsin showing an improvement in RMSD from 2.50 Å to 1.18 Å, halorhodopsin showing an improvement in RMSD from 1.84 Å to 1.48 Å, and bacteriorhodopsin showing an improvement in RMSD from 2.37 Å to 1.49 Å.

**Figure 2:**
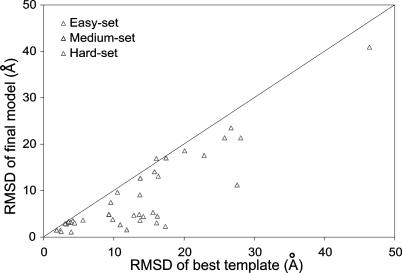
Application of TASSER to Membrane Proteins TASSER was applied to a benchmark set of 38 membrane proteins with structures in the PDB. RMSD to native for final models of TASSER versus RMSD to native for initial templates from PROSPECTOR_3. All points beneath the 45° line indicate an improvement in the TASSER model over the initial template. All template alignments with a sequence identity greater than 30% were excluded from consideration.

**Table 1:**
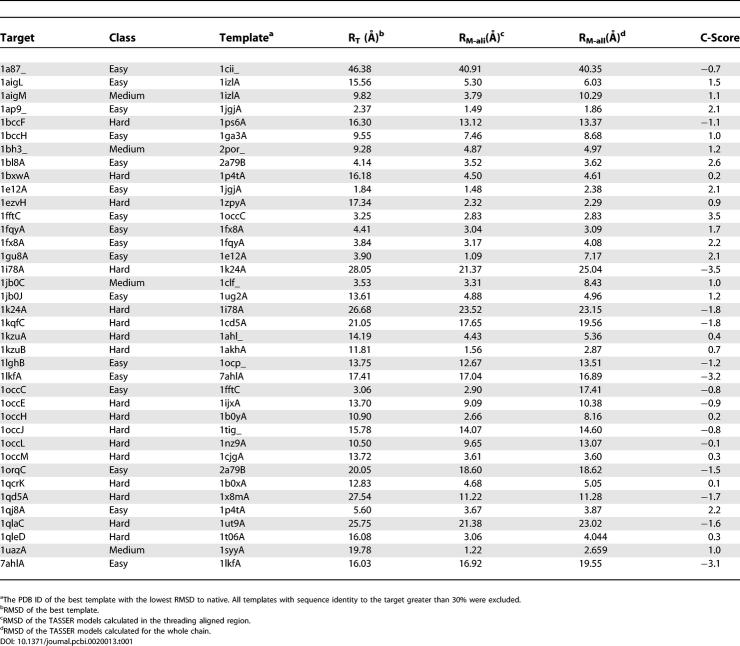
Modeling Result of the Benchmark Set of PDB Membrane Proteins

As indicated in Table 1, there is unfortunately no clear pattern with regard to the type of proteins where TASSER modeling will succeed, because its successes and failures are scattered among the different types of membrane proteins (including α- and β-proteins). In fact, there are two factors contributing to the success of TASSER modeling. First, the dominant factor is the correct identification of analog templates from the threading algorithm [14]. Reasonable threading alignments provide a good starting point and framework for the follow-up TASSER refinement. Second, the composite and optimized knowledge-based TASSER force field contributes to the refinement of the models. The result of the final predictions is a combination of complex threading and simulation procedures, which prohibits the induction of a simple and explicit rule for when TASSER will succeed. Nevertheless, most proteins with a TM helical topology were well modeled by TASSER, a feature that is important for GPCR modeling. This may be due to the well-constructed sequence profiles from the extensive set of helical proteins in the sequence database, because PROSPECTOR_3 partly relies on a profile-profile alignment and the TASSER potential uses the short-range correlations identified by sequence profile matches.

One of the difficulties in validating GPCR models is the paucity of experimental evidence that would provide a strong validation or invalidation of a given model. However, by providing a detailed benchmark of membrane proteins including seven-TM proteins and bovine RH itself, we have clearly demonstrated the ability of TASSER to refine membrane structures from low sequence–identity templates to structures that are closer to the native structure in an automated fashion. The automated nature of this approach offers a potential advantage over many other human expert–based methods that may introduce biases by a priori assuming specific structural characteristics or restraints.

### Sequence Clustering of Human GPCRs

Sequence analysis estimates that there are about 950 GPCRs in the human genome [3]. Combining the registered entries in the http://www.gpcr.org/7tm/htmls/entries.html and http://www.expasy.org/cgi-bin/lists?7tmrlist.txt databases (February 2004 release), we find a total of 907 human GPCR sequences less than 500 residues in length. To establish their evolutionary distance, we made an all-against-all sequence comparison and grouped them into clusters based on their sequence identities. Four hundred forty-six GPCRs belong to the same sequence cluster with greater than 30% sequence identity; 384 of these are olfactory receptors, the largest subfamily in class A GPCRs [1,2]. The second largest cluster has 38 GPCR sequences, of which half are chemokine receptors. Three hundred sixty-five GPCRs belong to 68 smaller clusters with two to 30 members, including the four-member cluster homologous to bovine RH. The remaining 58 GPCRs are orphans with no partners having sequence identity greater than 30%. If we use sequence cutoffs of 20%, 25%, 35%, and 40%, there are 664, 477, 377, and 308 members in the largest sequence cluster, respectively. These data demonstrate the high sequence (and therefore structure) diversity among the GPCRs. If the assumption is made that GPCRs should all contain seven-TM regions—which may be incorrect—better alignments should be constructed by identifying helical regions explicitly. However, these sequence diversity data strongly suggest that direct comparative modeling with the bovine RH structure alone is highly unlikely to capture the nature of the structural differences among GPCRs not only in the highly diverse loop regions but within the core TM regions, too.

### Threading Results

On threading the 907 GPCR sequences through our template library, a representative protein set covering PDB at the level of 35% sequence identity, PROSPECTOR_3 [14] assigns 778 sequences as easy targets, with average alignment coverage of 78%. This fraction of easy target assignment (about 86%) is significantly higher than in the PDB benchmark (about 67%) [20,21] and partly reflects the ability of PROSPECTOR_3 to detect the seven-TM helix bundle fold. One hundred twenty-nine sequences are assigned as medium/hard targets with average alignment coverage of 67%.

The average sequence identities between the target and template are 17.8% and 15.5% for the easy and medium/hard targets, respectively. Despite these low sequence identities, there is some correlation between easy/medium/hard assignments and the size of sequence clusters that partially reflects the sensitivity of the sequence profile term in PROSPECTOR_3. Among the 48 sequence clusters with three or more members, all members in 40 of the clusters are easy targets. The 129 medium/hard targets are populated in a few sequence clusters: 84 of 129 of the medium/hard targets are olfactory receptors in sequence cluster I, and 17 of 129 are orphan GPCRs.

For most easy targets (652/778), PROSPECTOR_3 hits at least one of four seven-TM proteins (Sensory Rhodopsin, Halorhodopsin, Bacteriorhodopsin, or Bovine Rhodopsin) as templates. Although further refinement of the core region and the ab initio prediction of the loop conformations are needed, these alignments provide a reasonable initial conformation for TASSER. In fact, even for proteins that do not hit these four templates, due to TASSER refinement, many are predicted to have the TM helix topology through a fully automated procedure. As shown below, there are 862 cases where the GPCR model has a typical TM helix topology but only 744 targets have these four TM helical proteins as starting templates.

### Confidence Score of Predicted Models

In Figure 3, the distribution of C-score, defined in Equation 1, for all 907 GPCRs is shown along with the corresponding C-score histogram of the PDB benchmark proteins [20]. Since the quality of threading templates is better for the GPCR proteins (reflected by the larger fraction of Easy targets and higher alignment coverage), many more GPCR models are populated at high C-scores.

**Figure 3:**
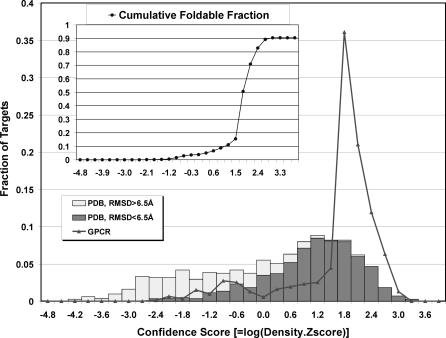
C-Score Distribution of the Predicted Models for the 907 GPCR Sequences The C-score histogram for the PDB benchmark proteins [20] is shown for comparison, where dark gray denotes those models with an RMSD less than 6.5 Å to native and the light gray those models whose RMSD greater than 6.5 Å. The C-score is defined as in Equation 1. Inset: The cumulative foldable fraction calculated under the assumption that the GPCR proteins have the same correlation between success and C-score as that of the PDB benchmark proteins.

In the PDB benchmark [20,21], for both globular and membrane proteins, there is a strong correlation between the C-score and the success rate of TASSER. For the 2,234 benchmark proteins ranging in size from 41 to 300 residues, the correlation coefficient between C-score and RMSD of the first model (corresponding to the most populated cluster) to native is 0.73. Of 38 membrane proteins in the benchmark, the correlation coefficient is 0.74, indicating that the TASSER confidence scoring system is directly applicable to TM proteins. The data in Figure 3 therefore imply that 819 of our GPCR models should have the correct topology. That is, for about 90% of the cases, at least one model in the top five predictions should have a core-region with an RMSD below 6.5 Å. There are 782, 749, and 698 cases with C-scores above 0.5, 1.0, and 1.5, respectively; in the benchmark, these C-scores correspond to a TASSER success rate of 94%, 97%, and 98%, respectively. Here, we note that a low RMSD just indicates the correctness of the overall topology of the helical arrangements. But the details of the loop regions and especially the ligand-binding sites may still be inaccurate. Further refinement at an atomic level as well as including the binding ligands in the modeling may be helpful.

It should be mentioned that although TASSER generates high confidence models for a substantial amount of medium/hard targets, the majority of the high C-score models are from easy targets. For example, among 749 targets with C-score greater than 1.0, 659 are from easy targets. This correlation indicates that although TASSER has the ability of structural refinement, the overall success still strongly relies on the quality of threading templates [34]. Furthermore, models generated with the explicit membrane potential showed little difference from those generated with the integrated form of TASSER (average TM score, 0.91; average RMSD, 1.8 Å).

### Conformational Changes from the Bovine RH Template

One of the major differences of the current approach from traditional CM methods is that TASSER refines the topology of threading alignments by rearranging the continuous fragments, while CM builds the model through optimally satisfying the restraints of the template structures. This results in the best CM models having the smallest variations from their initial template. Given the low sequence identity among GPCRs as a big family, one might expect significant differences from bovine RH, the only template available for CM methods. Thus, an interesting question is the extent to which TASSER has changed the conformation with respect to the initial template. In Figure 4A, we take all targets where TASSER employed bovine RH as an initial template and when the final model has a C-score greater than 1.3 and calculate the average distances of the residues of the final model from the corresponding residues in the bovine RH template according to the PROSPECTOR_3 alignment. On average, most residues in the TASSER model are greater than 4 Å away from the threading alignments with the maximum conformational changes in the loop and tail regions. In Figure 4B, we also show the helix angle changes of the predicted models with respect to bovine RH after superposition with TM-align [35]. Obviously, these conformational changes are significantly larger than the inherent resolution of TASSER modeling—as shown in the green triangles in Figure 4A and 4B; if we model bovine RH using its own crystal structure as the template, the overall RMSD of the model is 0.49 Å, with the observed variation along the RH template significantly smaller than the predicted average displacement for the other GPCR proteins. This degree of conformational change from the template is higher than could be expected by using a comparative modeling approach. Based on our previous benchmark and blind test results [20,21,36,37], most of the conformational deviations from the templates are in the correct direction toward native structures. For example, when starting from threading templates with a 4 to 5 Å RMSD to native, 58% of targets improve by at least 1 Å; when starting from a good threading template with a 2 to 3 Å RMSD to native, 43% of targets have at least a 0.5 Å improvement [20]. Even starting from the best structure alignments, similar improvements of final models relative to templates have been demonstrated (e.g., starting from initial structure alignments with an RMSD of 2 to 3 Å, 61% of targets have at least a 0.5 Å improvement) [36]. These data give us confidence that the observed deviations from the bovine RH template are most likely in the direction toward their native state.

**Figure 4:**
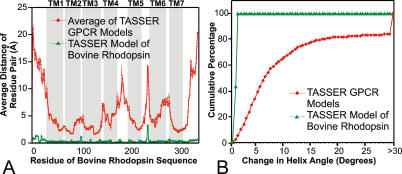
Conformational Changes of the Predicted TASSER Models from the Crystal Structure of Bovine RH Data are the average from those targets where bovine RH is a template with C-score greater than 1.3 (red diamonds). The green triangles denote the TASSER model for bovine RH when bovine RH itself is used as the template (ten missed residues in 1f88 are inserted in the TASSER modeling). This shows the inherent resolution of the TASSER model. (A) Average distance of each residue of the TASSER models from the bovine RH template along the sequence. TM helices are marked in gray. (B) Percentage of all helices with helix angle changes below the indicated thresholds.

### Number of TM Helices

Using an automatic procedure to identify TM helices by structurally aligning the models to a long helix, we can count the number of TM helices in the predicted models. Consistent with the cell membrane thickness, these are typically 17 to 25 residues long [38]. Among the 907 GPCRs, 740 have the seven-TM helix bundle topology. Ten GPCRs have eight long helices, where, as visually confirmed, the eighth helix is located in a tail outside the seven-TM helix bundle. We also checked by visual inspection all other 157 targets that have fewer than seven helices. Most have shorter sequence lengths and a regular TM-like topology. In general, these are truncated fragments of complete GPCR sequences [39,40].

There are 45 sequences whose global topology is not TM helix–like. Most have zero- to three-long, non-TM helices. Sixteen of these are incomplete or alternately spliced transcripts; most are missing the majority of their TM regions; three (Q8TDU0, Q8TDV3, Q96HT6) do not appear to be GPCRs at all based on sequence analysis [41]; two (Q9HC23 and P06850) are ligands misannotated as GPCRs [42,43]. The remainder may represent an incorrect TASSER prediction, since TASSER does not have a trustable C-score for many of these targets. In fact, only four of these targets have a C-score greater than 1, including a target misannotated as a GPCR (Q9HC23) and three sequences (Q16503, Q96HT6, and Q99997) that are fragments of N-terminal domains and do not include the RH portions of the target sequence [32].

### Structural Clusters of GPCR Models

Although there is little experimental evidence with which to directly test the validity of TASSER GPCR models, there exist indirect means of increasing the confidence in our predictions. First, an extensive membrane benchmark set from the PDB can be used to verify that TASSER can perform accurately on similar proteins. Second, we can check the self-consistency of the models under the assumption that closely related GPCRs or those with similar ligand specificities should in general adopt structures that are most similar to one another. To examine this, we first applied TM-align [35] to perform all-against-all structural alignments for the core regions of the predicted GPCR models and clustered the models based on their structural similarity. The average pairwise TM-score (a measure of fold consistency that ranges from 0 to 1, with 1 being identical and below 0.17 being random [44] and that should not be confused with TM regions) for all 907 targets is 0.71, with an average RMSD of 3.1 Å with over 82% alignment coverage. These data demonstrate the strong structural conservation of the characteristic seven-TM helix topology. The conformational variance arises mainly from differences in TM helix packing and local helix kinks (Figure 4). Using a high TM score cutoff of 0.95, 228 GPCRs are clustered into 35 clusters; all other GPCRs have no structural analogs at this high TM score cutoff.

In Table 2, we present the top ten cluster results, ranked by the number of cluster members. There is a very strong tendency for GPCR function conservation within a given structure cluster. For example, all 59 members in the first structural cluster are in the olfactory II family according to their Swiss-Prot assignments [39,40]. There is no olfactory GPCR in the second cluster; but all 51 members belong to class A (or putative class A) RH-like GPCRs. In the third cluster, all ten members are chemokine receptors, a subfamily of peptide receptors. This demonstrates a consistency of structures with similar function.

**Table 2:**
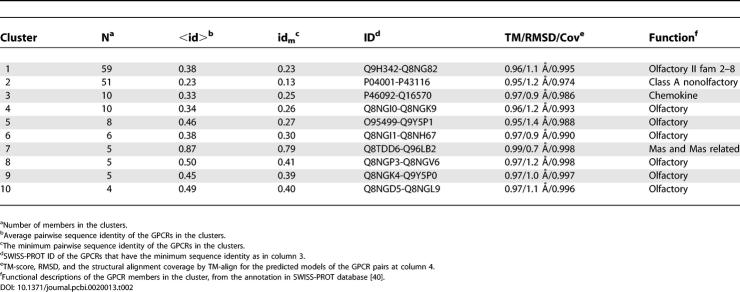
Top Ten Structural Clusters of GPCR Models

Interestingly, the degree of sequence conservation varies among the structure clusters. For example, in cluster 7 where all members are Mas or Mas-related receptors, the average pairwise sequence identity is 87%. In contrast, in cluster 2, the average sequence identity is 23%, much lower than the permissive threshold allowed for robust sequence-based function inference [45]. In cluster 2, the lowest pairwise sequence identity, between P04001-P43116, is 13%, but the models for these two GPCRs have a TM-score of 0.95 and an RMSD of 1.2 Å over 97.4% of the residues, consistent with the observation that structure is more conserved than sequence in evolution [46]. These examples of sequence divergence with structure convergence also appear in other clusters (Table 2). It seems suggestive that the global structural information in the GPCR models may be a useful complement to sequence-based functional analysis [6].

As an additional means of examining the consistency of the TASSER models, we examined whether the GPCR subfamily could be determined based on structure alone. A benchmark set of GPCR models with a C-score greater than 1.3 that were part of GPCR subfamilies with similar or identical ligand specificities was constructed including adenosine, angiotensin, chemokine, endothelial cell differentiation, galanin, melanocortin, opioid, P2Y nucleotide, prostaglandin, somatostatin, trace amine, and arginine vasopressin subfamilies. In total, the set consisted of 72 receptors and 12 subfamilies. N- and C-terminal tails were removed since TASSER tends to model these regions poorly. Each structure in the set was compared by TM-align to each other structure in the set. In 75% of the cases, the structure with the highest TM-score belongs to the correct subfamily (86% of cases have a correct subfamily member with the four highest scoring structures). While standard phylogenetic analysis of the peptide sequences alone would yield correct results (the average sequence identity between any structure and other members of its subfamily is 40.5%), this result does indicate a high degree of consistency amongst the TASSER model structures.

### Consistency of Models with Mutagenesis Studies

Although no solved X-ray or NMR structure is available for human GPCR sequences, numerous affinity labeling and site-directed mutagenesis experiments have been performed to identify critical residues and motifs that participate in ligand binding [2,31,47]. These data provide useful clues about the spatial contacts of the active site residues by which we can check to see if our models are consistent. We compared all TASSER models with C-scores greater than 1.3 to available mutagenesis studies including complement 5a receptor, thyroid-releasing hormone receptor, angiotensin receptor 1, adenosine 3 receptor, chemokine receptors, opioid receptors, formyl peptide receptor, thromboxane A_2_ receptor, neuromedin B receptor, melatonin 2 receptor, gonadotropin-releasing hormone receptor, and neuropeptide Y receptors [48–111]. A subset of these receptors is shown in Figure 5 with critical residues marked. Excluding N- and C-terminal tails, we have not found any data that would invalidate our TASSER models.

**Figure 5:**
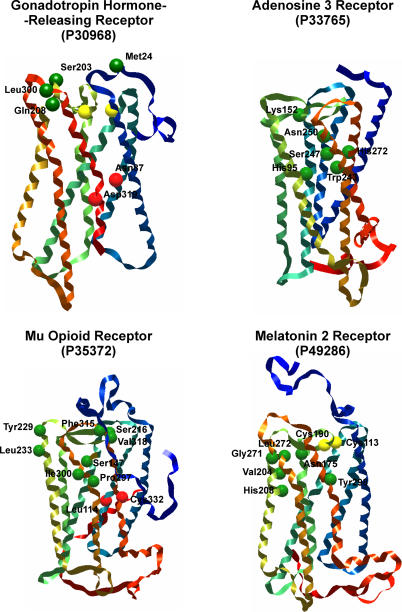
Consistency of Mutagenesis Studies with TASSER Predictions TASSER models for gonadotropin hormone-release receptor, adenosine 3 receptor, mu opioid receptor, and melatonin 2 Receptor are shown with experimental determined residue interactions highlighted as spheres (green, ligand binding; yellow, disulfide bond; red, residue-residue interaction).

### Prediction of Ligand Specificity for an Orphan Receptor

While the ligand binding affinities of GPCRs tend to closely follow the sequence-based phylogenetic placement, there are instances where this is not the case. One such instance is the RDC1 receptor. After being initially identified, RDC1 remained an orphan receptor for 15 years. While RDC1 does not have a striking homology to any other GPCR, it appears to be most closely related to the adrenomedullin receptor (ADMR) and places consistently with it in phylogenetic studies [112,113]. However, RDC1 was recently shown to be a receptor for the chemokine CXCL12, which also binds the chemokine receptor CXCR4. Pairwise comparison of the TASSER model for RDC1 to all other 906 GPCR models without N- and C-terminal tails (which were generated prior to the discovery that RDC1 binds CXCL12) yields CXCR4 as the highest TM-score receptor, despite having a lower sequence identity through the same region. In fact, 63 other models have a higher TM-score to RDC1 than ADMR, many of which are other chemokine receptors, suggesting common structural characteristics that distinguish chemokine and adrenomedullin receptors. It is important to note that this is strictly based on a direct structural comparison with no explicit attention paid to residue identities. Not only does this provide evidence strengthening the validity of TASSER models, but it also suggests that these structures may also be applied toward resolving the ligand specificities of orphan receptors as well as toward classification of weakly homologous GPCRs in other species.

## Discussion

By incorporating specific protein-membrane interactions into the TASSER force field, we have extended the TASSER threading-assembly-refinement methodology [20] and generated tertiary structure predictions for all 907 registered GPCRs in the human genome less than 500 amino acids long. Unlike traditional CM methods, TASSER does not require that the structures of homologous templates be solved, an essential attribute for the successful modeling of the whole set of human GPCR proteins, because most GPCRs have no close evolutionary relationship to proteins in the PDB.

Moreover, TASSER often refines the structures closer to native than the templates on which they are based [20,21]. This is particularly important for understanding the functional subtleties of the different classes of GPCRs when the models start from similar template alignments. These features have been demonstrated in the benchmark modeling of 38 representative medium-size membrane proteins from the PDB library, where TASSER has drawn the initial threading templates closer to native by an average RMSD of 4.9 Å in the threading aligned regions. An example of special interest is from RH of bovine rod outer segment membrane, the only solved GPCR protein. Excluding homologous proteins of sequence identity greater than 30% as well as bacteriorhodopsin, the threading program PROSPECTOR_3 [14] identifies three helical templates, all with global RMSD greater than 10 Å. After TASSER reassembly, the first model has a full-length RMSD to native of 4.6 Å, with the TM helix region having an RMSD of 2.1 Å. Recently, there have been many other attempts to model the tertiary structure of bovine RH. For example, Sale et al. [30] modeled the TM helix region using a statistical potential combined with 27 experimental distance constraints. They built a model with an RMSD of 3.2 Å to native in the TM region. Becker and coworkers [27,29] used PREDICT to model the TM region and generated a model whose TM region has an RMSD from native of 2.9 Å. Using MembStruk, Vaidehi et al. [114] built a model with an RMSD from native of 3.1 Å in the TM region and an RMSD from native of 8.3 Å for the full-length molecule. Compared with these results, the TASSER model is more accurate in the TM helix region, the loop/tail regions, and the full-length molecule.

Among the models generated for the 907 GPCR sequences, based on the confidence score established in comprehensive PDB benchmarking [20,21], 819 GPCRs are anticipated to have the correct global fold. Seven hundred fifty ORFs have the characteristic seven-TM helix topology, and 112 ORFs have the TM helix bundle topology with less than seven TM helices. There are 45 cases where TASSER generates non-TM helical models, primarily because these sequences come from periplasmic domain regions.

A quantitative structural comparison of the models from different GPCRs was performed by an all-against-all structural superposition. The average pairwise TM-score of the 907 GPCR models is 0.71, with an average 3.1 Å RMSD for 82% of residues in the core region. Using a restrictive TM-score cutoff of 0.95, the models tend to be grouped into structural clusters that have strong functional conservation, although sequences can be very divergent within the clusters. This is suggestive that structural information from the predicted GPCR models can be a useful complement to sequence-based functional analysis. It also demonstrates the robustness of TASSER, since structural convergence at low sequence identity is not built in but is a prediction.

A further validation of the predicted models includes structural consistency of GPCR subfamilies binding the same or similar ligands and consistency with mutagenesis studies. Furthermore, we demonstrate that the GPCR models can be more sensitive in determining ligand specificity than sequence-based methods, as is evidenced by the TASSER model of RDC1. Using sequence-based methods, RDC1 was expected to be an adrenomedullin receptor, since it shares its highest sequence identity and places phylogenetically with ADMR. However, RDC1 was recently shown to bind the chemokine CXCL12, whose only other known receptor is CXCR4. The TASSER model of RDC1 has as its closest structural neighbor, the model of CXCR4, further supporting the accuracy of TASSER models.

Comparative modeling approaches are useful in inferring the structures of sequences with greater than 30% sequence identity. They are also attractive because the computation resources required in generating these models are relatively small. However, 99% of GPCRs have a sequence identity less than 19.5% to the only solved GPCR structure, bovine RH. It is clear that comparative modeling alone would be unable to capture the range of structural diversity encompassed by the 907 receptors examined in this study. Alternatively, receptors can be modeled using specific restraints and assumptions that are assumed to be true for all GPCRs based on the solved rhodopsin structures at the risk of missing unforeseen structural characteristics of poorly characterized GPCRs. Threading using PROSPECTOR_3 along with TASSER has a demonstrated ability to construct accurate models of both membrane and globular proteins in a completely automated fashion with low-homology templates, thus providing an advantage over both the comparative modeling techniques and methods geared to strictly modeling GPCRs. While definitive validation of these structures is difficult given the paucity of clearly discriminating experimental evidence—the very reason why many have looked to predicted models in the first place—our benchmark studies of other membrane proteins and examination of the GPCR models for consistency with existing observations strongly suggests that these models are rather accurate.

The models presented here represent the most complete set of GPCRs models developed to date and offer a resource for ligand screening and other functional predications [27,115]. Given the extensive computational time required to generate these models (several decades, if run on a single processor), this study makes a resource available for experimental testing that would be infeasible for most experimental labs to generate independently. All predicted GPCR models, as well as follow-up functional analysis data, are available for noncommercial users on our Web site (http://www.bioinformatics.buffalo.edu/GPCR).

## Materials and Methods

### Template identification.

For a given GPCR sequence, we run the threading program PROSPECTOR_3 to identify putative related template structures in the PDB. PROSPECTOR_3 is an iterative sequence/structure alignment approach [14]. On the basis of the score significance and the consensus of template alignments, proteins are categorized into easy, medium, and hard targets. These terms refer to the relative confidence in the accuracy of the predicted threading models. According to the benchmark, 80% of the threading-predicted alignments for the easy targets have an RMSD to native less than 6.5 Å in the aligned regions [20]. This alignment accuracy is essentially the same for both globular and membrane proteins [21]. For the medium/hard targets, the topology of the template is often correct, but the global alignment can be in error. Nevertheless, the local fragments from the template alignment can be utilized as building blocks in TASSER [20].

### Substructure/fragment assembly.

Continuous fragments (more than five residues) are excised from the five top scoring threading templates for the easy targets and up to the 20 top templates for the medium/hard targets. For the GPCR sequences, these fragments are mainly long TM helices that will be reassembled under the guide of the TASSER force field that consists of an optimized combination of a reduced knowledge-based potential [17] and consensus contact restraints from threading [14]. The loops connecting the helices are generated by the TASSER ab initio structure prediction procedure.

Conformational space is searched by the parallel hyperbolic Monte Carlo algorithm [116]. Depending on GPCR length, 40 to 80 replicas are used with larger molecules having more replicas. Two kinds of major conformational updates are implemented: Off-lattice movements of the template-excised fragments involve rigid translations and rotations controlled by the three Euler angles of each fragment. Lattice-confined loop residues are subject to two to six bond movements and multibond sequence shifts [17].

### Extension of TASSER to membrane proteins.

The hydrophobic interactions in the original TASSER force field are applied only to the loop/tail residues, which are assumed to be outside the membrane. For the putative TM helices, because of the hydrophobic membrane environment, a propensity for hydrophilic side chains to face to the interior of the protein is included. TM helices are assigned from PSIPRED [117]. We also tried other TM predictors, e.g., MEMSAT [38], but the differences are small. In general, the local geometry of a template-derived substructure remains similar to that in the template [20]. However, considering the variance of helix shape and the presence of local kinks along the TM helices, we allow a small bending deformation for the aligned TM helices. A strong penalty potential term of *E* ~ Δ*RMSD* ^4^ is employed (the form of the fourth power is somewhat arbitrary but was chosen based on trial and error); Δ*RMSD* denotes the RMSD between the excised template substructure and the deformed substructure in the simulations.

### Model selection and assessment.

Trajectories of the 14 lowest temperature replicas are submitted to SPICKER [37] for structure clustering. SPICKER first identifies a center structure that has the most neighboring structures within an RMSD threshold *R*_cut_. The first cluster is defined as a group of structures including the center structure and all its neighbors. The second cluster is similarly defined after all the structures in the first cluster have been removed, etc. *R*_cut_ is defined in an iterative way: The initial *R*_cut_ is set to 7.5 Å. If the structures are too tightly distributed, *R*_cut_ will gradually decrease until the first cluster contains less than 70% of the total number of structures or until *R*_cut_ is 3.5 Å. If the structures are too divergent, *R*_cut_ will gradually increase until the first cluster includes more than 15% of structures or until *R*_cut_ = 12 Å.

To avoid distortions of clusters from disordered tail variations, a two-step clustering is implemented: We first run SPICKER on the structurally well defined core (the putative TM region based on PSIPRED) and the tail regions separately; then, we dock the conformations of the three separate parts (the two tails and the core) into the final full-length model based on the superposition of these regions onto the structure obtained by clustering the full-length conformations. Reduced models (C_α_s and side-chain centers of mass) are provided from the clustered structures. Backbone and side-chain heavy atoms are added using PULCHRA [118].

The final models are ranked and assessed on the basis of the confidence score [20]:


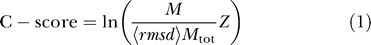


where *M* is the multiplicity of structures in a SPICKER cluster, *M*_tot_ is the total number of structures submitted for clustering, and 〈*rmsd*〉 denotes the average RMSD of the structures relative to the cluster centroid. The logarithm in Equation 1 serves to expand the range of the C-score distribution of the predicted structures. If we define a correct fold as the one with an RMSD to native below 6.5 Å [119], the PDB benchmark results [20] show that for all targets with a C-score threshold of −0.5, the total false positive/false negative rate is 12.4%/14.7%.

## Supporting Information

### Accession Numbers

The Swiss-Prot (http://www.ebi.ac.uk/swissprot) accession numbers for the four sequences of registered human GPCRs that have a sequence identity to bovine RH greater than 30% are P03999, P04000, P04001, and P08100.

The Protein Data Bank (http://www.rcsb.org/pdb) accession number for the bovine RH crystal structure is 1f88A.

## Abbreviations

ADMR: adrenomedullin receptor
CM: comparative modeling
GPCR: G protein–coupled receptor
NMR: nuclear magnetic resonance
PDB: Protein Data Bank
RH: rhodopsin
TASSER: threading assembly refinement
TM: transmembrane
