# Correction: Structure Modeling of All Identified G Protein–Coupled Receptors in the Human Genome

**DOI:** 10.1371/journal.pcbi.0020029

**Published:** 2006-02-17

**Authors:** Yang Zhang, Mark E DeVries, Jeffrey Skolnick

DOI: 10.1371/journal.pcbi.0020013

In *PLoS Computational Biology,* volume 2, issue 2:

The URL provided for the GPCR model database in the published article is no longer active. The database is now located at http://cssb.biology.gatech.edu/skolnick/files/gpcr/gpcr.html.

